# Historical Statement of the Galvanic Discovery, and of the Publications That Have Appeared on That Subject

**Published:** 1802-02

**Authors:** 


### *Historical Statement of the Galvanic Discovery, and of the Publications that have appeared on that Subject.*

[ Continued from our laſt, pp. 70—73. ]

MR. GALVANI likewiſe found, that different metals joined together in a different manner, were the moſt efficient, but above all ſilver. The experiment ſucceeded under water quite as well as in open air. It ſurpriſed Mr. Galvani, that if the hook faſtened in the ſpinal marrow was touched, the water replacing the conducting arch would excite contractions in the animal which did not enſue on the experiment being made under oil. Our author thought himſelf now entitled to aſſert, that there is in the animal a double electricity, oppoſite to one another; the one in the muſcles, the other in the nerves, or both at the ſame time in either of them. In order to aſcertain this circumſtance, he coated the nerve or the ſpine of the back with tin-foil, by which he found the motions to be extremely increaſed. The contractions however proceeded leſs vigorouſly by coating the muſcle. If a part of the nerve as well as of the muſcle was coated with a non-conducting ſubſtance, as ſilk or pitch diſſolved in oil, no contractions were excited, even on applying the conducting arch. Mr. Galvani alſo obſerved that this animal electricity, as it was ſtyled by him, opens itſelf an eaſy paſſage through ſome conducting ſolid bodies; whereas, through others it paſſes with more difficulty; but the beſt conductor of this electricity is water, while oils deſtroy the phenomena of this matter. To the conducting property of water, the author was inclined to aſcribe the fact, that having coated the ſpinal marrow, and ſeparated the lower extremities, he only obſerved contractions in the leg that was touched, which were however communicated to the other, as ſoon as he had brought both in contact with each other. Mr. Galvani proves next, in the moſt convincing manner, that the contractions are no wiſe excited by mechanical ſtimuli, as was firſt ſuppoſed by others. He alſo obſerved the contractions to ſucceed leſs vigorouſly when the muſcles were placed on a glaſs pannel, and the ſpinal marrow on an electric plate, but they became ſtronger after having changed that ſituation; moſt violently, however, they were excited, when the legs as well as the ſpinal marrow were placed on coated pannels, particularly on giving them ſome electrical ſtrokes. When the nerves were entirely ſeparated from the ſurrounding parts the contractions conſiderably increaſed. The experiments ſucceeded equally well with warm blooded and cold blooded animals. The contractions being diminiſhed, minifhed, were reftored to their former vigor after a time of reft had been allowed.

We muft acquiefce in mentioning only the conjectures and conclufions which the author has drawn from his difcovery, without farther entering into the fubtle arguments he has brought forth in fupport of his ideas. The conclufions he builds on the facts related by him, are as follow: Animals are endued with a peculiar electricity, to which he gives the appellation of *animal electricity*, and which he thinks to be contained in moft animal parts, chiefly however manifefting itfelf in the mufcles and nerves. It feems to be fecerned in the brain from the blood, whence it is communicated through the nerves to the different parts of the body, but it appears to refide chiefly in the mufcles. A mufcular fibre is fimilar to a Leyden phial, and the nerve reprefents the conductor of the phial, and confequently the whole mufcular fubftance is to be confidered as a number of Leyden phials. The external furface of the mufcle poffeffes negative, the internal fubftance pofitive electricity. The interior of the nerves is compofed of a matter capable of conducting electricity, while the exterior prevents by the oily coating its effufion and difperfion. Mufcular motions proceed, when the electric fluid is conducted from the interior of the mufcle into the nerve, whence it is brought back again to the mufcles, either through the external fluid of the nerves, or through the membranes and the adjacent parts, as it were through an arch, fo that, according to the laws of equilibrium, the fame quantity may be united in the negative electric part of the mufcular fibre, which iffued by means of the ftimulus in the nerves from the pofitive electrical part. Dr. Eufebius Valli relates, in his Memoir on Animal Electricity, a feries of thirty-two experiments, by which he particularly endeavoured to determine the effects of tobacco and opium on Galvanifm, but he found thefe fubftances, as well as other poifons, to have no action on the electrical principle; the gafes, however, do violently affect it.

In a letter from Mr. Boffano Carminati to Mr. Galvani, the following notice is given of the refults of Mr. Volta's experiments. "Prepared frogs, whofe fpinal marrow and part of the nerves are coated with metallic plates, may be ufed as the moft fenfible electrometers. The negative, and not the pofitive electricity, is feated in the nerves." Mr. Volta defcribes the experiment from which he has drawn that conclufion, in a letter to Dr. Boronio. "Not being able to trace the nature of this very weak electricity by means of the moft fenfible electrometer, I proceeded in another manner. I called to my mind, that two phials being brought in contact with each other by their their ſynonymous electrical ſurfaces, do not diſcharge themſelves, which however is the caſe, as ſoon as they touch each other with their oppoſite electrical points, and I thought it therefore not indifferent, whether I applied the internal coating of a weakly charged phial to the muſcle or to the nerve. On making a ſeries of experiments according to this idea, I have frequently obſerved, that on applying the poſitive ſurface of a phial to the nerve, $\frac{5}{100}$ or $\frac{6}{100}$° of the electrometer, did ſuffice for producing contractions in a frog, whereas hardly from $\frac{20}{100}$ to $\frac{30}{100}$° were ſufficient for the ſame purpoſe, when the poſitive ſurface of the Leyden phial was brought in contact with the muſcle, and the negative ſurface with the nerve. Thence we may conclude, that a negative electricity is imparted by Nature to the nerve, and a poſitive electricity to the muſcles." Mr. Galvani, in reply to this, writes in a letter, addreſſed to Mr. Carminati, as follows: "Could we not think it probable, that in the caſe where the head of the Leyden phial was brought in combination with the nerve, the convulſions might have entirely or for the moſt part been owing to the electrical matter having penetrated from the internal coating of the phial into the nerve, and thence into the internal ſurface of the muſcular fibre? For though, according to my hypotheſis, a plus of electricity exiſts in the head of the phial as well as in the interior of the muſcular ſubſtance, both electricities are moſt probably efficient in the ſame degree of force; and it is moſt likely that the weak natural electricity of the nerves is overcome by the ſtronger artificial electricity. We may therefore aſſert, that a part of the poſitive electricity of the phial penetrates into the internal ſubſtance of the muſcles by means of the conducting ſubſtance of the nerves, where, being aſſimilated to their natural electricity, it occaſions a diſcharge, which is not produced by a proceſs of the animal machine, but effected by the Leyden phial in the hands of the experimenter."

[ To be continued. ]

### *On Medical Degrees.*

[ Continued from our laſt, p. 13. ]

